# Mercury-sensitive water channels as possible sensors of water potentials in pollen

**DOI:** 10.1093/jxb/ert311

**Published:** 2013-10-05

**Authors:** Bruria Shachar-Hill, Adrian E. Hill, Janet Powell, Jeremy N. Skepper, Yair Shachar-Hill

**Affiliations:** ^1^Multi-Imaging Centre, Cambridge University, Cambridge, UK; ^2^Department of Physiology, Development and Neuroscience, Cambridge University, Cambridge, UK; ^3^Department of Plant Biology, Plant Biology Building, Michigan State University, East Lansing, MI 48824-1312, USA

**Keywords:** Cell bursting, cell walls, mercury inhibition, osmosensors, pollen cells, water channels.

## Abstract

The growing pollen tube is central to plant reproduction and is a long-standing model for cellular tip growth in biology. Rapid osmotically driven growth is maintained under variable conditions, which requires osmosensing and regulation. This study explores the mechanism of water entry and the potential role of osmosensory regulation in maintaining pollen growth. The osmotic permeability of the plasmalemma of *Lilium* pollen tubes was measured from plasmolysis rates to be 1.32±0.31×10^–3^ cm s^–1^. Mercuric ions reduce this permeability by 65%. Simulations using an osmotic model of pollen tube growth predict that an osmosensor at the cell membrane controls pectin deposition at the cell tip; inhibiting the sensor is predicted to cause tip bursting due to cell wall thinning. It was found that adding mercury to growing pollen tubes caused such a bursting of the tips. The model indicates that lowering the osmotic permeability *per se* does not lead to bursting but rather to thickening of the tip. The time course of induced bursting showed no time lag and was independent of mercury concentration, compatible with a surface site of action. The submaximal bursting response to intermediate mercuric ion concentration was independent of the concentration of calcium ions, showing that bursting is not due to a competitive inhibition of calcium binding or entry. Bursting with the same time course was also shown by cells growing on potassium-free media, indicating that potassium channels (implicated in mechanosensing) are not involved in the bursting response. The possible involvement of mercury-sensitive water channels as osmosensors and current knowledge of these in pollen cells are discussed.

## Introduction

Pollen tubes carry the male nuclei by a unique mechanism, replacing the need for motile sperm in all spermatophytes with the partial exception of the primitive gymnosperms (cycads and *Ginko*). The tubes grow by a tip extension mechanism that involves the construction of a domed tip by vesicle-mediated deposition of wall material that is spread by a high internal turgor pressure. Growth of the tube involves the uptake of water and the synthesis of cytoplasmic volume and osmotic agents at high rates, putting pollen tubes amongst the fastest growing cells in the plant kingdom. The dynamics and control of tube growth have close affinities with the growth of fungal hyphae, and there is overlap and exchange of concepts and modelling between these areas. Tip growth is a similar process in root hairs.

For tube growth, speed is important for reproductive success, and in angiosperms it is limited by its competence in growing after germinating on a suitable stigmatic surface. Of prime importance in pollen growth is the ability to grow with a stable wall thickness and turgor under changing osmotic conditions which involves some form of osmosensing, This is not confined to plants, but is faced by animal sperm which apparently have an essential osmosensor that controls cell stability and function after osmotic stress (Chen and Duan, 2011; Chen *et al.*, 2011). The protoplasm of the pollen tip is covered by a viscous polymer liquid film when initially deposited, and this has to be spread by expansion driven by turgor pressure; in this respect, tip growth of the tube is quite different from the much slower expansion growth of most plant cells. The wall thickness in the growing region is remarkably constant, as is the turgor pressure (Benkert *et al.*, 1997; Hill *et al.*, 2012). Changes in osmotic pressure differences driving water into the cell must be matched by the ability of the cell tip to control its wall thickness or the cells may burst or stop growing. Such control requires osmosensing. In the following, this term is used for sensing (ΔΠ–Δ*P*) the driving force for water or difference in thermodynamic water potential across the plasma membrane.

The field of osmosensors now encompasses a fast-expanding number of possible candidates for the molecular basis of ‘osmoreception’ in many cell types. These are usually understood to be integral membrane proteins. First, there are possible osmoreceptors which detect an osmotic pressure difference across a membrane or in the phase bounding one side of a membrane. Examples are osmotically sensitive ion channels (Zimmerberg and Parsegian, 1986; Jiang *et al.*, 2003) and osmo-sensitive transporters (Racher *et al.*, 1999; Wood, 2011). In addition, there are mechanosensitive or ‘stretch’ channels and molecules which are implicated in osmosensing as a proxy, for example responding to swelling or shrinkage by stretching the lipid membrane bounding the cell or cell organelle (Poolman *et al*., 2002, 2004; Reiser *et al.*, 2003; Schliess *et al.*, 2007; Hammami *et al.*, 2009; Schaber *et al.*, 2010; Maathuis, 2011). Finally there are the molecules identified by genetic analysis such as vanilloid receptors (Liu *et al.*, 2006; Cohen, 2007) and histidine kinases (Schumacher *et al.*, 1997; Urao *et al.*, 1999; Razani *et al.*, 2003; Wohlbach *et al.*, 2008; Meena *et al.*, 2010) which have not been characterized mechanistically as primary receptors of stretch or osmotic pressure but may be the first components in a cell signalling cascade linked to them. In particular, stretch-activated Ca channels have been found in tip protoplasts of *Lilium* (Dutta and Robinson, 2004) and are associated with Ca influxes, but, although obviously important, these are not primary osmosensor candidates.

It has also been suggested, in a review of the role of aquaporins (AQPs) in plants, animals, fungi, and bacteria, that these molecules act as sensors of both osmotic and turgor pressure differences across membranes (Hill *et al.*, 2004), being located at the plasma membrane and internal membranes of organelles such as the tonoplast of plant cells. The relevant membrane for sensing the osmotic driving force of pollen cells must be the plasma membrane which is subject to combined osmotic and hydrostatic forces driving water through the water-filled core of a channel which would serve as a localized region in the membrane responsive to a change in water potential between the cell and its environment. In certain animal systems, an association between an AQP and a signalling pathway has been indicated (Liu *et al.*, 2006; Benfenati *et al.*, 2011).

Modelling in conjunction with experiments is a powerful tool for studying tip growth because the complex role of turgor pressure in both water entry and tip expansion during steady-state growth, combined with the interaction with most of the other parameters of the cell, soon becomes too difficult to handle in any other way. The position has been recently reviewed in some detail (Zonia, 2010; Zonia and Munnik, 2011; Kroeger and Geitmann, 2012*a*, *b*; Kroeger *et al.*, 2012) and the physiological models may be grouped under different headings: (i) tip ‘morphodynamics’; these models involve the expansion of the tip wall by pressure to give the shape and growth patterns observed by quantitative microscopy (da Riva Ricci and Kendrick, 1972; Bartnicki-Garcia *et al.*, 2000; Dumais *et al.*, 2004, 2006; Geitmann, 2006, 2010; Campas and Mahadevan, 2009; Geitmann and Dumais, 2009; Geitmann and Ortega, 2009; Fayant *et al.*, 2010); (ii) vesicle supply models involving the cytology of the tip region and the supply of wall material (Gierz and Bartnicki-Garcia, 2001; Tindemans *et al.*, 2006); (iii) ionic models involving the known pumps and transporters of the cell and their possible role in generating oscillations of current flow (Liu *et al.*, 2010); and (iv) models generating oscillatory growth rates in pollen tubes (Dumais *et al.*, 2006; Kroeger *et al.*, 2008; Yan *et al.*, 2009; Zerzour *et al.*, 2009; Liu *et al.*, 2010; Rojas *et al.*, 2011).

Modelling studies can be used to suggest whether sensors are required and, if so, what they are most likely to sense and control for the stability of important measured variables—what may be called their input–output relationship. In a recent paper on tip growth, the extension rate, and stability of pollen cells of *Lilium longiflorum*, an osmotic model of the growing cell was presented in which the osmotic and turgor pressures created by the steady-state accumulation of solute within the cell was coupled to the deposition of pectin at the tip apex followed by its rapid expansion by the turgor (Hill *et al.*, 2012). This model, supported by experiments and results taken from the literature, was initially developed to encompass the relevant physicochemical variables into a connected scheme: the number of possible interconnections and parameters was already too complex to allow simple reasoning to be effective.

The model was deliberately kept as simple as possible to capture the essence of osmotically driven pollen tip growth in the context of the whole cell, and to allow testing of hypotheses about regulation. It incorporates well-known physicochemical relationships in which the growth rate is controlled by the permeability, the osmotic difference, the turgor, and the deposition rate of pectin, all of which are interconnected. The turgor pressure both drives tip growth and is itself a function of the growth rate. However, for stable growth, an osmosensor in the membrane is required, which detects the driving force on water between the cytoplasm and the medium and controls the rate of pectin deposition at the tip.

In this study, physiological evidence for such a sensor in the tube plasmalemma is provided. It is shown (i) that when bursting is inhibited by hypertonic media, mercury (Hg) causes a substantial fall in the osmotic permeability (*P*_os_) of the plasmalemma; (ii) that in normal media Hg ions cause rapid and progressive bursting of pollen tubes at their tips; (iii) that in simulations with the model bursting cannot simply be caused by reducing *P*_os_ because this acts to inhibit bursting in tubes; (iv) that when bursting is inhibited Hg does not affect cell integrity or cytoplasmic streaming; and (v) that within the duration of experiments Hg ions are not causing bursting by competing with Ca ions or blocking K channels. In the Discussion, the osmo-turgor sensor required by the osmotic model is revisited, and it is shown that it is in general agreement with what has been found in these experiments.

## Materials and methods

### Pollen

Flowering stems of *L. longiflorum* were obtained from florists and kept in water at room temperature. Pollen was collected from anthers 2 d after dehiscing. Pollen was used fresh or stored at –20 °C after 2h drying at room temperature. Stored pollen was re-hydrated in a humidified atmosphere in Petri dishes lined with wet filter paper at room temperature for 1h before use. No difference in growth rates or morphology could be seen.

### Growth

Pollen tubes were grown in the germination medium (see below) in the following ways. Medium solidified with 1% agarose on cavity slides for growth measurements and for following the effects of 500 µM BAPTA [1,2-bis(*o*-aminophenoxy)ethane-*N*,*N*,*N*′,*N*′-tetraacetic acid]. For experiments to measure osmotic permeabilities the flow chamber was used. Pollen was then grown in fluid medium on cover slips coated with 400 µg ml^–1^ poly-d-lysine (mol. wt 70–150kDa). For confocal microscopy, pollen was grown in plastic Petri dishes of 35mm diameter with a cover slip base. The glass was coated with poly-d-lysine at 400 µg ml^–1^ or a thin layer of 1% agarose. The range of growth rates was the same in fluid and solid media.

### Media

Basic ionic medium comprised KCl 1mM, CaCl_2_ 0.1mM, H_3_BO_3_ 1.6mM, MES/TRIS 5mM, pH 5.5–5.6. Basic growth medium comprised basic ionic medium plus sucrose 300mM. Osmotic pressure was varied by changing the sucrose concentration, and Hg was added as HgCl_2_. For growth media lacking K ions, the basic growth medium with 300mM sucrose had (i) KCl removed (medium S) or (ii) KCl removed and 300mM PEG 400 (polyethylene glycol 400) replacing the sucrose (medium P). Growth rates after germination were determined from tube lengths.

### Flow chamber

The flow chamber and accessories (suitable for imaging using a confocal microscope) were purchased from Warner Instruments (Harvard). Large 250 µm gaskets were used as spacers. Flow was gravimetric from 20ml syringes. Tubing, a chamber perfusion manifold, and valves were used to regulate the flow rate at 0.4ml min^–1^.

### Microscopy

Pollen grown on solid medium in cavity slides or on cover slips for the flow chamber were used to follow growth and the effects of varying conditions on growing tubes using a Zeiss light microscope fitted with a Leica digital camera connected to a computer, and Leica AS software was used for analysis.

### Electron microscopy

Lily pollen tubes were grown in the media listed above either in fluid form or solidified with agarose. Pollen tubes were fixed in 4% glutaraldehyde in 0.1M PIPES buffer for 1–2h at room temperature, rinsed in 0.1M PIPES buffer three times for 5min, and post-fixed in 1% osmium ferricyanide for 1h. They were rinsed in 0.1M PIPES buffer three times for 5min, bulk stained with uranyl acetate in maleate buffer for 1h, and rinsed in water. They were then dehydrated in 70% ethanol three times for 5min, 95% ethanol three times for 5min, and 100% ethanol three times for 5min, in acetonitrile twice for 5min, 50% acetonitrile/50% Quetol 651 resin for 1h, 25% acetinitrile/75% Quetol 651 resin overnight, 100% Quetol 651 resin (with no BDMA) for 1 d, and finally 100% Quetol 651 resin+BDMA for 2 d. They were then embedded and cured at 60 °C for 24h.

### Sectioning and staining

Sections were cut at 70nm on a Leica Ultramicrotome with a Diatome diamond knife, collected on 100 mesh copper grids or Formvar-coated copper slot grids, counterstained with uranyl acetate and lead citrate, and viewed in a Phillips CM100 transmission electron microscope.

### Plasmolysis experiments

The osmotic permeability was determined by measurements of tip plasmolysis as in a previous report (Hill *et al.*, 2012). When subjected to a hypertonic osmoticum in a flow chamber, the cytoplasm retracted initially at the tube tip


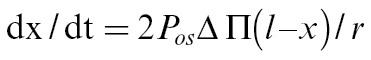
(1)

where *x* is the retraction length, *r* the tube radius, ΔΠ the osmotic pressure difference, and *P*_os_ the osmotic permeability. When the retraction attains a plateau, *x=l*, the length of the osmotic zone at the tip. A diagram of the process with an experimental result can be seen in Supplementary Fig. S3 available at *JXB* online. The retraction rate d*x/*d*t* in mid-range (i.e. close to *x*=*l/*2) and the tube radius were calculated from video stills and used to calculate *P*_os_ for each tube.

### Simulations

The osmotic model was implemented using Mathematica 4.0. on desktop computers with a set of parameters and independent variables closely matching those known for growing *Lilium* pollen tubes (see Hill *et al.*, 2012 for details). Unknown parameters were assigned magnitudes which resulted in dependent variable values close to those observed in normal growth, and this comprised the standard parameter value set of the model. After an initial steady state was reached, this was perturbed by a change in a parameter value or by a relationship between particular variables, according to the objective of the simulation. The model equations were advanced iteratively by time steps of 0.4 s. Smoothing functions (exponentials with suitable time constants) were used to control (i) abrupt perturbations in parameters; (ii) sensor feedback; and (iii) the rate of expansion of the tip area by the pressure which could otherwise cause artefactual fluctuations in growth rate and pressure at the frequency of the iterations. The smoothing half-times used were between 0.2 and 0.5 of the computational time step.

Hg treatment was simulated (i) in a sensor model by stepping the active sensor density in the plasmalemma to zero (complete inhibition) and simultaneously reducing the osmotic permeability *P*_os_ to 36% of its initial value, as found in plasmolysis experiments, or (ii) in a model variant with no sensor (but a constant value of tip pectin extrusion equal to that in the steady state with sensor) and reducing the osmotic permeability *P*_os_ to 36% alone. In all cases, bursting occurs when the tip film thickness becomes very small, approaching the critical thickness τ set as ≤20nm.

## Results

### Water permeability is reduced by mercury

**Fig. 1.**
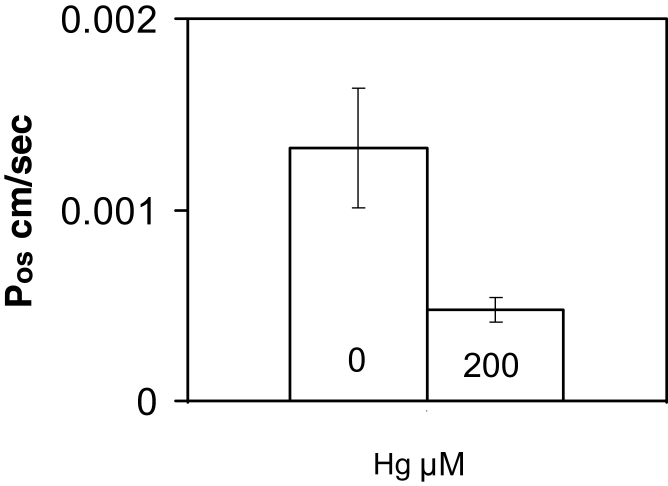
Measurement of the osmotic permeability of *Lilium* plasmalemma by tip plasmolysis with and without Hg; 64% of the osmotic permeability is inhibited by 200 µM HgCl_2_ (SD error bars).

When *Lilium* tubes were bathed in a hypertonic medium containing sucrose (800mM), plasmolysis occurred from the tip backwards. This confinement to the tip is because the osmotically permeable part of the tube is restricted to a short region near the apex (Hill *et al.*, 2012). Measurement of the rate of cytoplasmic withdrawal at the tip was used to estimate the osmotic permeability *P*_os_ (see the Materials and methods). The value obtained by this method was 1.32±0.31×10^–3^ cm s^–1^—very similar to that derived from the swelling of isolated *Lilium* tube protoplasts (i.e. lacking the cell wall) (Sommer *et al.*, 2007, 2008). Addition of HgCl_2_ at 200 µM to the hypertonic medium lowers the value of *P*_os_ to 0.48×10^–3^ cm s^–1^ (Fig. 1); thus Hg reduced the exposed cell membrane osmotic permeability to about a third of its normal value. This indicates that Hg acts at the cell surface and that its primary action is to inhibit a water channel. It suggests that such a channel has aquaporin-like properties; indeed members of the AQP family are widespread in plants (Chrispeels *et al.*, 2001; Maurel *et al.*, 2009).

### Cell bursting induced by mercury

**Fig. 2.**
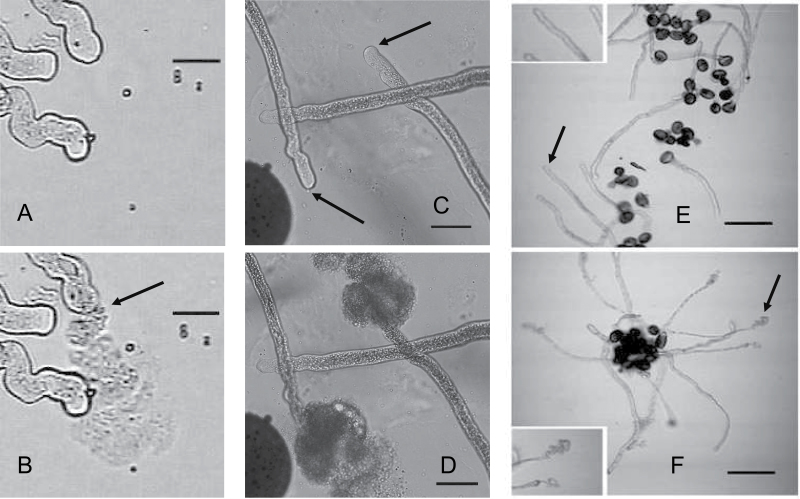
Bursting of pollen cells 1–2min after flooding pollen tubes growing in agar with Hg solution (100 µM). *Mahonia* (bar=20 µm): (A) +Hg prior to bursting; (B) +Hg, top cell bursts ejecting a large plume of cytoplasm from a small area of tip apex in 0.5 s. (arrow). *Lilium* (bar=30 µm): (C) +Hg, showing two cells prior to bursting (arrows). (D) +Hg, showing two tip plumes of cytoplasm ejected in <1 s. *Arabidopsis* (bar=100 µm): (E) +Hg. prior to bursting showing smooth intact tips (arrow and inset ×2); (F) +Hg, bursting cells after 1–2min showing tip plumes (arrow and inset ×2).

**Fig. 3.**
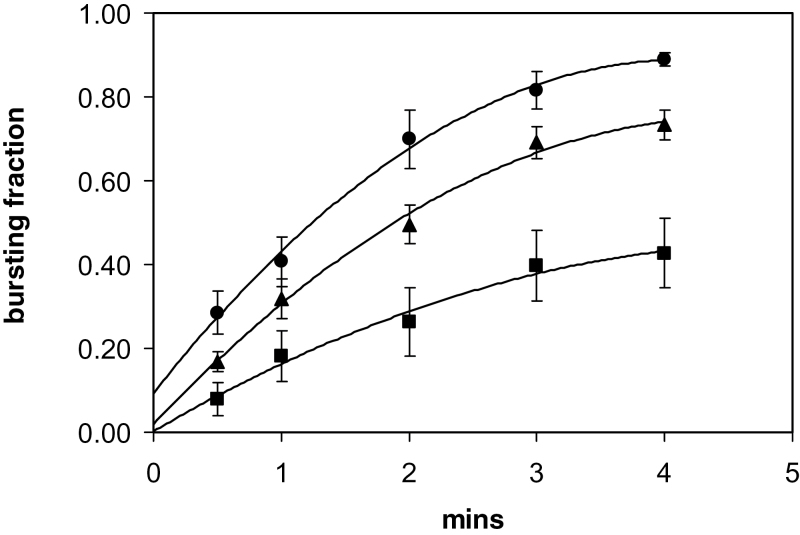
Time course of Hg-induced bursting in *Lilium* (Ca 100 µM); 25 µM Hg (squares), 100 µM Hg (triangles), and 200 µM Hg (circles). The data are fitted to second-order polynomial curves with ± SD error bars and *R* ^2^ >0.99 for each curve. Note the [Hg] dependence and the absence of lags. The curves are extrapolated to zero time. In most experiments, there is a small amount of bursting before the addition of Hg.

**Fig. 4.**
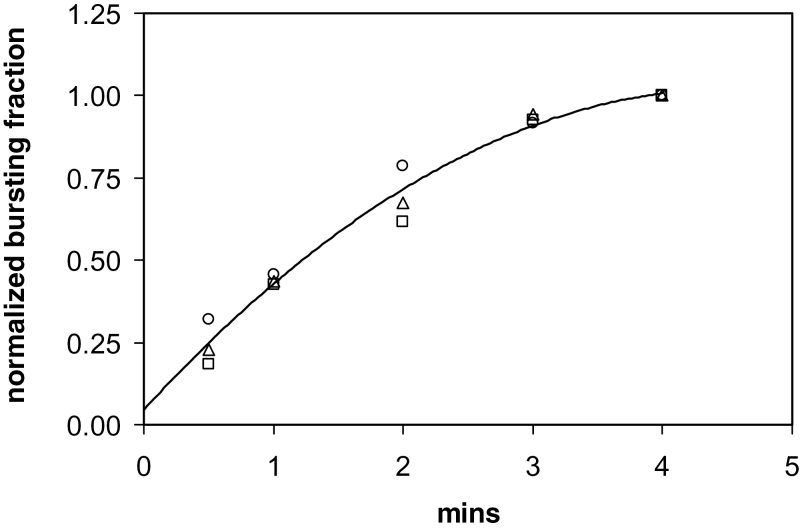
Time course of Hg bursting; each data set is normalized to the bursting fraction at 4min. The curve for all data points is a second-order polynomial with *R* ^2^=0.98. Hg concentrations: 25 µM (squares), 100 µM (triangles), 200 µM (circles). The time courses of the three component data sets are similar.

When Hg ions were added to pollen tubes growing *in vitro*, there was an instantaneous onset of bursting. This phenomenon was observed in *Lilium*, *Mahonia*, and *Arabidopsis* pollen (Fig. 2). Using *Lilium* pollen tubes growing on surface agarose, there was a time-dependent bursting of the cell tips as measured between 0.5min and 4.0min. The bursting fraction at any time was also concentration dependent as measured with Hg concentrations of 25, 100, and 200 µM (Fig. 3). When the bursting fractions are normalized to that at 4min, it can be seen that they follow the same time course, and a curve-fit for the total data set shows no sign of a time lag at the onset (Fig. 4).

### Bursting in other media

**Fig. 5.**
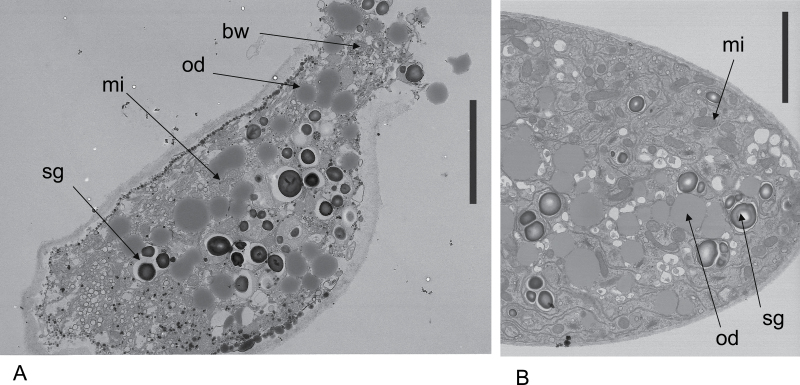
Transmission electron microscopic sections of *Lilium* pollen tubes. (A) Bursting tube near the tip showing ejection of cell contents after Hg. The organelles are intact and similar to those of growing tubes. (B) Growing tube without Hg showing cell contents. Bw, bursting wall; od, oil droplet; mi, mitochondrion; sg, starch granule. Bars=5 µm.

*Lilium* pollen germinated and grew on agarose in media lacking potassium ions. Along with normal growth medium (see the Materials and methods), two other media were prepared: (S) similar to normal medium with 300 mOs sucrose but without K; and (P) with 300 mOs PEG 400 replacing sucrose but also without K. In these, tubes grew for many hours, sometimes overnight, with morphology, streaming, and growth rates very similar to those in normal growth medium (Supplementary Fig. S1A at *JXB* online). When challenged with 200 µM HgCl_2_, they all showed similar bursting fractions (Supplementary Fig. S1B). These results indicate that Hg inhibition of K channels or the uptake of K for use as a solute (or any membrane system utilizing external sucrose) were not the cause of bursting. Energy and solutes for osmotic pressure would be generated from internal starch or lipid reserves, as seen in Fig. 5.

### Hg-treated cells appear normal

Although Hg leads to bursting, this must be a specific reaction. (i) Light microscopy video imaging of cells subjected to Hg showed vigorous cytoplasmic streaming right up until the time of tip bursting which, as can be seen from Fig. 3, took up to 4min. The growth and streaming was interrupted by the bursting reaction, not by any apparent damage to cell structures (Supplementary Video S1 at *JXB* online). (ii) Cells in which bursting has been prevented by raising the external osmotic pressure with 0.8M sucrose, as in the plasmolysis experiments described above, showed no adverse reactions to Hg ions as judged by microscopic observation; whilst plasmolysis was occurring at the tip, the cells looked normal and exhibited vigorous cytoplasmic streaming (Supplementary Videos S2, S3). (iii) Although it is a comparatively rare event to catch a bursting tip by electron microscopy, it is possible, and cell organelles are clearly identifiable in (Fig. 5) and similar to those of steadily growing tubes.

### Competition with Ca

**Fig. 6.**
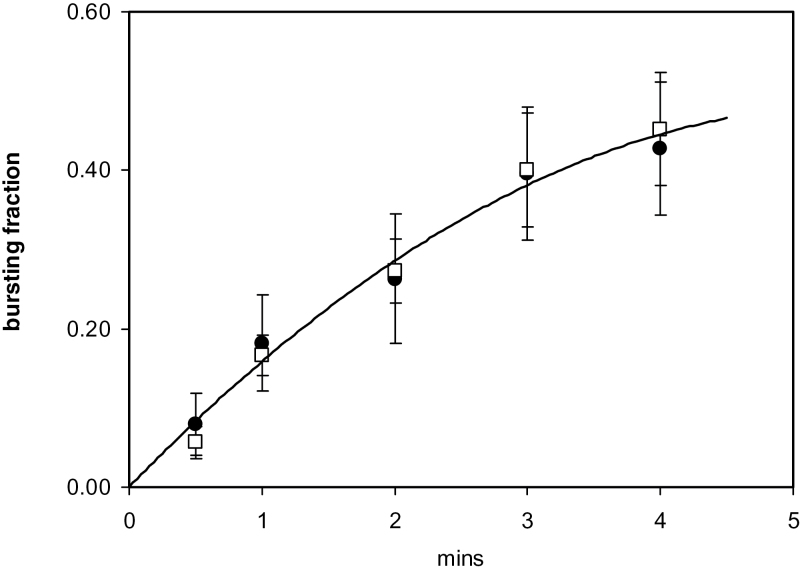
Similar Hg-induced bursting curves at low and high Ca levels show that Hg does not interact with Ca binding. Hg 25 µM; Ca 100 µM (circles), Ca 2000 µM (squares). SD error bars and all points fitted to a second-order polynomial curve, *R* ^2^=0.98.

It has been shown that removal of Ca from the bathing solution causes bursting in *Lilium* (Messerli and Robinson, 1997) which is consistent with its role of cross-linking pectin as well as being an essential ion for normal growth when a transmembrane Ca current carries this ion into the cell (Hepler and Winship, 2010). The fractional bursting when tubes growing on agarose containing 100 µM Ca were superfused with a zero-Ca saline containing the Ca-complexing agent BAPTA at 500 µM reached 50% in just over a minute (Hill *et al.*, 2012). To test whether Hg could be competing with Ca for entry sites, bursting was tested with suboptimal concentrations of Hg (25 µM) in the presence of either 100 µM (normal) or 2000 µM Ca. The results are shown in Fig. 6 where it is apparent that raising Ca by 20-fold to the high concentration of 2mM made no difference to the bursting pattern in *Lilium* tubes. This indicates that Ca and Hg do not compete for binding sites.

### Modelling predicts that inhibiting osmosensing should cause cell bursting

**Fig. 7.**
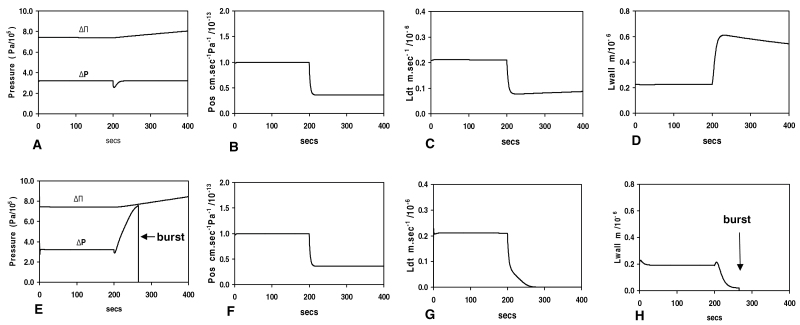
Results from model simulations of bursting. At 200 s, the established steady-state growth is subject to a step-change representing the effects of adding Hg. (A–D) Model without sensor feedback but with a decrease of *P*_os_ to 36% as found in experiments. (A) The turgor pressure Δ*P* shows a perturbation and then stabilizes at its former value. The osmotic pressure ΔΠ of the whole tube slowly rises as solute accumulation continues during a slower growth rate. (B) Osmotic permeability *P*_os_. (C) the growth rate *L*d*t* slows. (D) The tip wall thickness *L*_wall_ increases. (E–H) Model with sensor feedback, the step-change now comprising both a reduction of sensor feedback to zero and a decrease of *P*_os_ to 36%. (E) Cessation of wall production causes the turgor pressure Δ*P* to rise towards the osmotic pressure value as water comes into equilibrium with the external medium. but when the cell bursts it falls to zero. (F) Osmotic permeability *P*_os_. (G) growth rate *L*d*t* falls to zero (arrow) due to the sensor inhibition shutting down wall extrusion. (H) The tip wall thickness *L*_wall_ decreases to a critical value (*L*_wall_ <0.02 µm, arrow) and the cell bursts.

It has previously been postulated that water channels could serve as osmosensors (Hill *et al.*, 2004). If Hg inhibits a water channel protein which is also acting as a sensor, it should both inhibit the sensor pathway and decrease the osmotic permeability *P*_os_. In a model simulation (Hill *et al.*, 2012), it is possible to examine these effects independently, as shown in Fig. 7.

An osmotic model simulation was run without sensor feedback (i.e. with pectin extrusion at a constant rate). After reducing *P*_os_ to 36% to mimic the effect of Hg found here, the computed growth rate fell by a similar amount, representing slowed water entry; at the same time, the wall thickness rose by ~300% (Fig. 7A–D), consistent with reduced growth but constant pectin extrusion; this indicates that bursting would be inhibited. The same simulation but with sensor feedback operative during the reduction in *P*_os_ (Fig. 7E–H) together with inhibition of the signal from the sensor controlling pectin extrusion, caused rapid bursting; pectin extrusion was interrupted and the wall thinned to bursting point at the critical thickness.

## Discussion

### The effects of Hg on cells

Hg has a surface effect on many plant, animal, and bacterial cells by binding to thiol residues in proteins. Because Hg can be toxic to organisms and can affect cellular systems, it is important to consider its effects on other preparations. Many of its recognized toxic effects are related to its entry into the circulation in the animal body, and this aspect will not be considered here. Hg^2+^, when applied at high concentration, can interact with lipid bilayers by changing membrane fluidity and binding to certain lipid sites (Delnomdedieu and Allis, 1993; Suwalsky *et al.*, 2000; Garcia *et al.*, 2005; Le *et al.*, 2009). Lipid bilayers are permeable to HgCl_2_ as a neutral molecule (Gutknecht, 1981) but, where Hg^2+^ ions are concerned, rapid effects are confined to its action on membrane proteins; external application of HgCl_2_ only results in binding when thiol groups in hydrophilic clefts have surface access to the medium (Kiss and Osipenko, 1994; Krajewska, 2008). In all these interactions, there is no basic destabilization of the bilayer, and no bursting.

The most frequent use of Hg in physiology is to examine its effect on water transport where extensively applied to roots (Wan *et al.*, 2004; Ionenko *et al.*, 2006, 2010, 2012; Zhou *et al.*, 2007; Dustmamatov and Zholkevich, 2008; Ehlert *et al.*, 2009; Devi *et al.*, 2012) and other cells (Henzler and Steudle, 2000), and in animal systems it has been used in numerous preparations, for example in *Xenopus* oocytes (Barnes *et al.*, 1999), epithelia (Patil *et al.*, 2001; Castillo *et al.*, 2005; Philip *et al.*, 2008), and diverse cell types (Voigtlaender *et al.*, 2002; Gao *et al.*, 2005; Heo *et al.*, 2008; Ismail *et al.*, 2009; Campbell *et al.*, 2012; Lee *et al.*, 2012). In all these systems, other measurements on the cells involved are made subsequent to Hg binding for some time afterwards, involving different aspects of water movement: swelling, osmotic permeability, and hydraulic conductivities, among others. These would not be possible if the cells burst or their membranes became generally leaky.

Osmotic flow across a membrane into the cell is given by the general equation


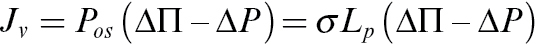
(2)

where the osmotic permeability *P*_os_ is the product of a reflexion coefficient σ and a hydraulic conductance *L*_p_, and ΔΠ and Δ*P* (turgor) are the osmotic and pressure differences between cell (i) and medium (o); that is, (Π_i_–Π_o_) and (*P*_i_–*P*_o_). If Hg were to render the membrane seriously porous, the value of σ would fall to zero, as would *P*_os_; that is, osmosis across the membrane would come to a halt. This cannot apply to plasmolysis at the *Lilium* tip described here because Hg treatment leaves an osmotically functioning membrane.

### The site of Hg action and its time course

In the bursting described here, Hg is binding to a surface site and this is likely to be activating or inhibiting a cell signalling pathway. which apparently controls the exocytosis of pectin at the apical tip disc (Hill *et al.*, 2012). The time courses of bursting in Figs 3 and 4 show that the process increases monotonically without any apparent lag, and approaches a plateau. These three curves are clearly homologous, as shown in Fig. 4. The bursting fraction at the different times relative to 25 µM Hg are 1:1.85 (SD 0.18):2.54 (SD 0.66), but these are not proportional to the external Hg concentration ratios, which are 1:4:8, showing that the binding is saturating.

It is important to consider whether the bursting curves represent simply the loading of the cytoplasm with Hg. If this were so, given the permeability *P*_Hg_ of lipid bilayers to the neutral HgCl_2_ species of ~10^–2^ cm s^–1^ (Gutknecht, 1981) and the cylindrical dimensions of the *Lilium* tube (radius *r=* ~8 µm in these studies), the half-time for loading the cell, considered as a single compartment, is given by


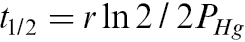
(3)

which would be of the order of 0.02 s. However, the observed half-time, as can be judged from Figs 3 and 4, is about a minute. Clearly, the bursting must be due to other processes which have an overall time course 2–3 orders of magnitude greater. This does not mean that Hg entry into the cytoplasm does not occur, but that it is not rate limiting.

It is possible that Hg could be binding at two different sites, one initiating bursting and the other inhibiting the water permeability *P*_os_. However, this is unlikely for the following reason. It was shown as the result of model simulations (Results section) that inhibition of *P*_os_ affects the bursting process by progressively inhibiting bursting; the fact that the curves of Fig. 3 superimpose so well indicates that if two sites were involved they would, in general, have different binding constants and saturate differently as the Hg concentration is raised. Homology of curves—over an 8-fold rise in Hg concentration—would not be likely. If there is one site involved, reducing the osmotic permeability, it must be at the plasma membrane.

### Bursting fraction

It is apparent that the curves, although rising with Hg concentration, are approaching plateaus and there is a fraction of cells which do not burst (Fig. 3). If cells are left for longer experimental periods—up to an hour—this fraction is stable. It is instructive to understand why this is so, and here the model (Hill *et al.*, 2012) is used to examine the bursting process in more detail.

The model treats the extreme tip as a liquid film of pectin which, after extrusion by the cell, is simultaneously hardened by chemical cross-linkage and thinned by the turgor pressure which stretches it. Bursting is due to the interruption of pectin extrusion, with the result that the film rapidly thins towards a thickness where it may become unstable and burst. Most liquid films under a variety of conditions are not stable below a critical thickness (τ) of 0.02 µm (Coons *et al.*, 2005; Manev and Nguyen, 2005). Simulations with the pollen model show that if the osmosensor is progressively inhibited by Hg—together with a Hg-sensitive reduction of *P*_os_—the result is eventual thinning of the tip wall to zero. When a cell is subjected to partial inhibition, the tip wall thins and stabilizes at a new value, which may be above or below τ. Unless τ is set to a finite value, simulation indicates that the tube will never burst until the inhibition is fully 100% because there is nothing to prevent the cell continuing to grow, albeit slowly, with an extremely thin tip. In simulations, a value of τ=0.02 µm has been used (Supplementary Fig. S2A at *JXB* online), being 10% of the normal tip thickness of 0.2 µm as shown by transmission electron microscopy (Hill *et al.*, 2012).

The value of most parameters in the model affect the final wall thickness: the degree of Hg inhibition θ, the permeability *P*_os_, the tube radius *r*, sensor control of the rate of wall deposition *k*, and the amount of sensor resident in the membrane β. The rate of pectin deposition is then proportional to (1–θ)β*k* where θ is a function of the Hg concentration and its binding constant. In any population of tubes used in an experiment, there will be a distribution of these parameters—indeed permeability measurements on single tubes show a range of values (Fig. 1)—and the cluster present in any cell will determine its sensitivity to bursting. This can be simulated with variation in a single parameter. When β was varied by 50%, partial bursting was observed (Supplementary Fig. *S2B* at JXB online). The Hg binding constant to the sensor-channel site and the distribution of various parameters in a real cell are not known, but it can be assumed that they will generate the plateaus of Fig. 3. The time course of these curves must be dependent upon the kinetics of the signalling cascade between (1–θ)β*k* and the pectin extrusion, which may be regarded (as a first approximation) to be independent of the amount of functioning sensor (1–θ)β and the output signal *k*, which explains the homology seen in Fig. 4.

To summarize: the homology between the curves and the fact that they have no lag period (i.e. the initial slopes are positive at *t* = 0) is consistent with the following interpretation: (i) the Hg is acting at the cell membrane, which accords with the fact that Hg inhibits a substantial fraction of the surface membrane water permeability, *P*_os_; (ii) raising the Hg concentration over the range 25–200 µM leads to increased Hg binding to the receptor; (iii) bursting is the result of a signalling pathway the amplitude of which depends on the amount of Hg binding; and (iv) variations of cell parameters lead to some cells being more sensitive and some more resistant to partial Hg inhibition. As the Hg concentration is raised, the bursting fraction approaches 100%.

### A water channel can meet the requirements for a sensor of water potential gradients

If a channel is permeable to water but excludes solutes, it represents a semi-permeable element of length *L* spanning the lipid bilayer. The channel constitutes a volume inaccessible to the solutes (Zimmerberg and Parsegian, 1986) with the additional property that it spans the membrane from the exterior to the cytoplasm. There will be differences in osmotic and (in plants) turgor pressure which are reflected in a gradient of pressure *P*_ch_ in the channel


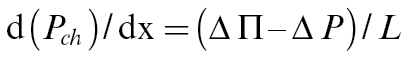
(4)

which will be zero when there is no difference of thermodynamic water potential across the membrane (ΔΠ=Δ*P*) as in a cell at equilibrium. When there is a transmembrane difference, there will be a gradient of pressure within the channel which could deform the channel protein, depending on its molecular architecture, and act as a sensor of both the magnitude and direction of the driving force on the water (Hill *et al.*, 2004). The channel would have to interact with an associated signalling sequence in the membrane. It is of interest that AQPs have been found in functional or structural association with the vanilloid ‘stretch receptor’ TRPV4, a putative osmoreceptor in membranes (Liu *et al.*, 2006; Benfenati *et al.*, 2011) and they have also been directly implicated as osmo- and stretch receptors themselves (Murakami *et al.*, 2006; Chen and Duan, 2011; Chen *et al.*, 2011).

### Aquaporins and pollen membranes

The results suggest that an AQP-like molecule is present in the pollen plasmlemma. If it is a true AQP, it should appear in inventories of AQP molecules expressed in pollen cells. It is known that the two AQPs AtTIP1;3 and AtTIP5;1 are strongly expressed in *Arabidopsis* pollen (Soto *et al.*, 2008), but neither sequence homology nor fluorescence tagging indicates a surface localization. A search for members of the PIP subfamily in *Arabidopsis* pollen has so far drawn a blank (Yamada *et al.*, 2011). However, measurements on *P*_os_ in protoplasts extracted from *Lilium* pollen have shown that the value for ungerminated grains is only half that of the pollen tube (Sommer *et al.*, 2008), which indicates that water channels are inserted into the plasmalemma during growth. In *Arabidopsis*, a member of the NIP-subfamily, AtNIP7;1, is expressed in late stages of pollen development but has little water permeability compared with that for boric acid, urea, and glycerol (Li *et al.*, 2011). In *Nicotiana*, NtPIP1;1 and NtPIP2;1 RNAs accumulate in pollen and pollen tubes, albeit at a low level; NtPIP2;1 has AQP activity but NtPIP1;1 does not (Bots *et al.*, 2005). It would seem that much more work needs to be done on pollen MIPs and MIP-like proteins before this can be resolved.

## Supplementary data

Supplementary data are available at *JXB* online.

Figure S1. Growth and bursting of pollen germinated and grown on media without K ions.

Figure S2. Simulations of bursting for different levels of Hg binding to the sensor molecule (inhibition) using the osmotic model of the lily pollen tube.

Figure S3. Diagram and data of the tip retraction process

Video S1. Four bursting tubes after application of 100 µM HgCl_2_ at the start.

Video S2. Plasmolysing tube 1 in 0.8M sucrose and 100 µM HgCl_2_ showing streaming.

Video S3. Plasmolysing tube 2 in 0.8M sucrose and 100 µM HgCl_2_ showing streaming.

Supplementary Data
