# Supplementary material for: Mercury-sensitive water channels as possible sensors of water potentials in pollen
Source: J Exp Bot. 2013 Oct 5;64(16):5195–205. doi: 10.1093/jxb/ert311 (PMC3830494; doi:10.1093/jxb/ert311)
Supplement: Supplementary Data [file supp_64_16_5195__index.html]

Mercury-sensitive water channels as possible sensors of water potentials in pollen — Mercury-sensitive water channels as possible sensors of water potentials in pollen — Supplementary Data 

# Mercury-sensitive water channels as possible sensors of water potentials in pollen

## Supplementary Data

Data files

**Files in this Data Supplement:**

- Supplementary Data - Supplementary Data
- Supplementary Data - Supplementary Data
- Supplementary Data - Supplementary Data
- Supplementary Data - Supplementary Data
