# Supplementary material for: Mercury-sensitive water channels as possible sensors of water potentials in pollen
Source: J Exp Bot. 2013 Oct 5;64(16):5195–205. doi: 10.1093/jxb/ert311 (PMC3830494; doi:10.1093/jxb/ert311)
Supplement: Supplementary Data [file supp_ert311_jexbot104281_file001.pdf]

J. Exp. Botany. ‘Mercury-sensitive Water Channels as Possible Sensors of Water Potentials in Pollen’.
Bruria Shachar-Hill, Adrian E. Hill, Janet Powell, Jeremy N. Skepper, and Yair Shachar-Hill.

Figure S1

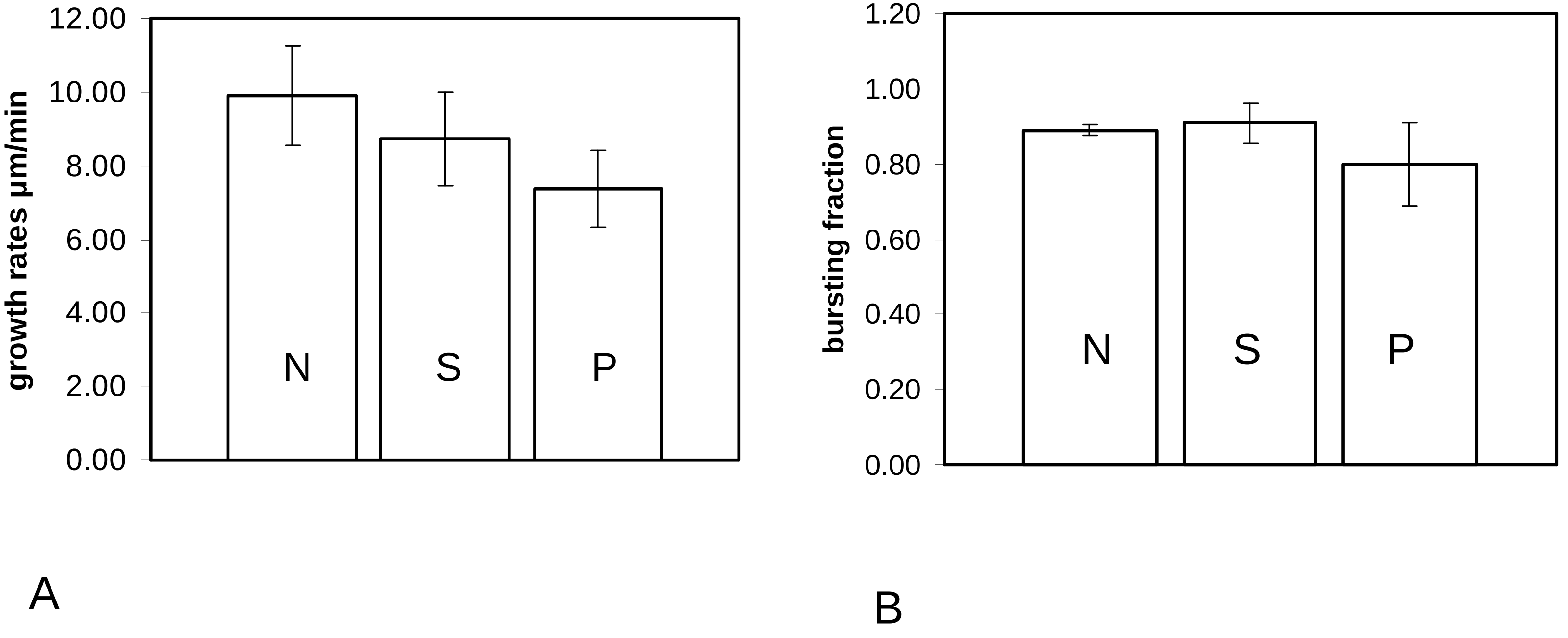

Growth and bursting of pollen germinated and grown on media without K ions.
A. Linear growth rates. **B**. Bursting fractions using 200µM Hg. N – normal growth medium; S – without K; P – without K and PEG 400 replacing sucrose (see Materials and methods: Media).

Figure S2

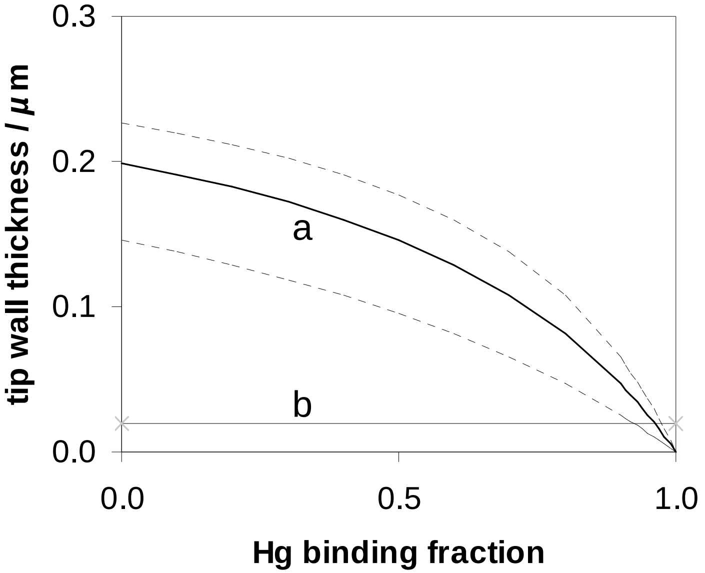

A

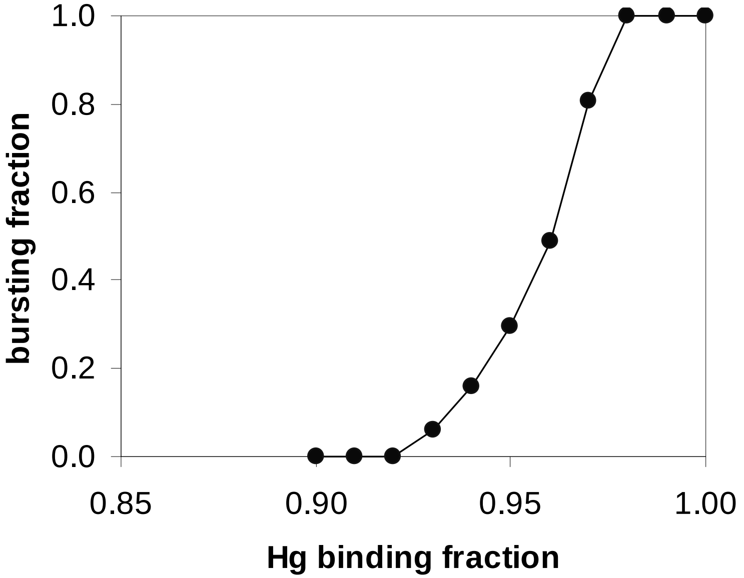

B

Simulations of bursting for different levels of Hg binding to the sensor molecule (inhibition) using the osmotic model of the lily pollen tube (Hill *et al* 2012 *PLoS ONE* **7**, e36585).
A. a – thinning curve of the tip cell wall over the range of Hg binding from 0% to 100% using standard lily parameters; the tip thins from 0.2 µm to zero; flanking this curve (dotted lines) are curves with 50% more (upper) or 50% less (lower) sensor in the membrane; b – level set for bursting (the critical thickness) at 0.02 µm. **B**. Fraction of cells that burst, plotted from A. It can be seen that bursting only begins above 90% Hg-binding and the fraction of bursts approaches 100% as the binding increases i.e. there is partial bursting in the tube population over this range.

Figure S3

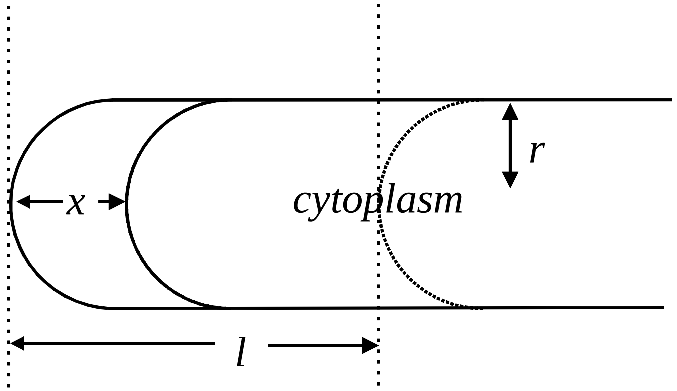

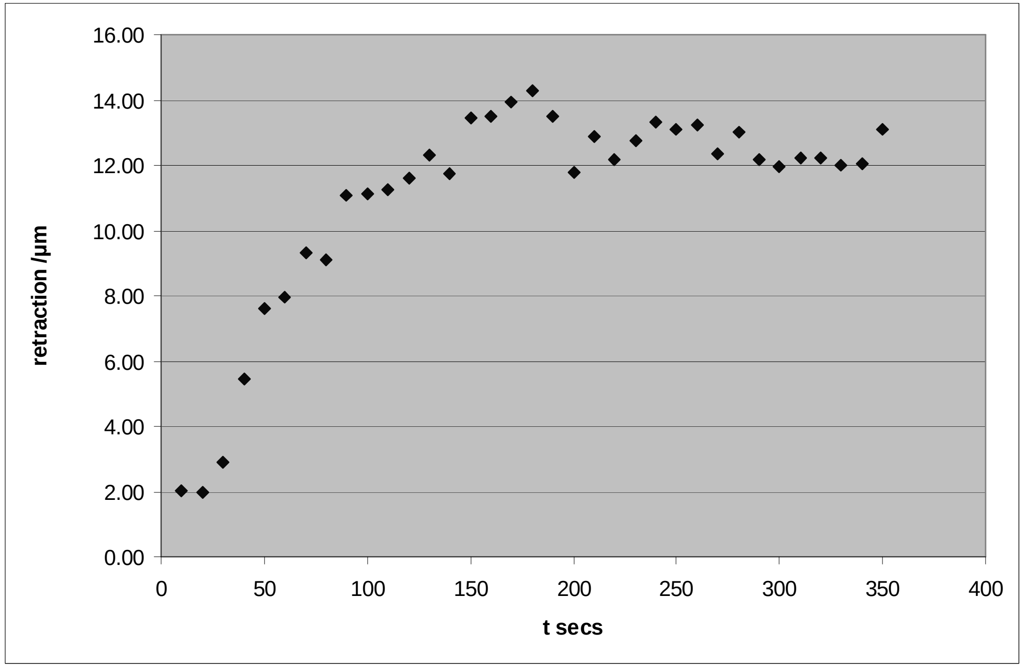

A

B

Diagram and data of the tip retraction process. **A**. During plasmolysis, which is initially confined to the tip, the retraction of cytoplasm in a tube of radius ***r*** with time proceeds to a plateau at ***x*=*l***. **B**. A typical sample of the process.
